# TRAJECTORIES OF QUALITY OF LIFE AMONG FRAIL OLDER ADULTS IN KOREA: THE ROLE OF SOCIAL NETWORK

**DOI:** 10.1093/geroni/igae098.2775

**Published:** 2024-12-31

**Authors:** Yeonsoo Shin, Giyeon Kim

**Affiliations:** Chung-Ang University, Seoul, Republic of Korea; Chung-Ang University, Seoul, Republic of Korea

## Abstract

The purpose of this study was to examine the trajectories of quality of life among frail older adults in Korea, with a specific focus on the role of social networks on quality of life. Using data from waves 1 (2006) to 6 (2016) of the Korean Longitudinal Study of Ageing (KLoSA), a total of 3,177 older adults aged 65 and older at baseline was included in analyses. This study analyzed the trajectories of quality of life in all subjects, as well as frail and non-frail groups. Furthermore, the study investigated the effect of social networks on both the intercept and slope of quality of life. Results from multiple-group latent growth curve modeling showed that the intercept of quality of life for the frail group was lower than that of the non-frail group. While quality of life showed a declining trajectory for both the overall sample and the non-frail subgroup, the frail subgroup exhibited an increasing trajectory. Moreover, participation in social activities positively affected the quality of life intercept for both frail and non-frail older adults. Interestingly, quality of life among frail older adults living alone was found to be increasing. These findings provide valuable insights into the nuanced dynamics of quality of life among frail older adults in Korea. The study underscores the importance of promoting participation in social activities among this population and suggests further research focusing on frail older adults living alone.

